# Delete and survive: strategies of programmed genetic material elimination in eukaryotes

**DOI:** 10.1111/brv.12796

**Published:** 2021-09-20

**Authors:** Dmitrij Dedukh, Alla Krasikova

**Affiliations:** ^1^ Saint‐Petersburg State University 7/9 Universitetskaya Embankment Saint‐Petersburg 199034 Russia

**Keywords:** asexual hybrids, B chromosomes, chromatin diminution, chromosomal lagging, micronuclei, programmed DNA elimination, sex chromosomes

## Abstract

Genome stability is a crucial feature of eukaryotic organisms because its alteration drastically affects the normal development and survival of cells and the organism as a whole. Nevertheless, some organisms can selectively eliminate part of their genomes from certain cell types during specific stages of ontogenesis. This review aims to describe the phenomenon of programmed DNA elimination, which includes chromatin diminution (together with programmed genome rearrangement or DNA rearrangements), B and sex chromosome elimination, paternal genome elimination, parasitically induced genome elimination, and genome elimination in animal and plant hybrids. During programmed DNA elimination, individual chromosomal fragments, whole chromosomes, and even entire parental genomes can be selectively removed. Programmed DNA elimination occurs independently in different organisms, ranging from ciliate protozoa to mammals. Depending on the sequences destined for exclusion, programmed DNA elimination may serve as a radical mechanism of dosage compensation and inactivation of unnecessary or dangerous genetic entities. In hybrids, genome elimination results from competition between parental genomes. Despite the different consequences of DNA elimination, all genetic material destined for elimination must be first recognised, epigenetically marked, separated, and then removed and degraded.

## I. INTRODUCTION

Eukaryotic cells maintain and ensure their genome's stability, thereby allowing development of the organism and assuring the transmission of genetic material to offspring. Further, the genetic material of an individual needs to be stable during its transmission to the progeny. Some organisms, ranging from ciliate protozoa to mammals, nevertheless possess complex mechanisms to eliminate selectively some of their genetic material during specific stages of ontogenesis (Tobler, 1986; Dawley & Bogart, 1989; Kloc & Zagrodzinska, 2001; Burt & Trivers, 2009; Schön, Martens & van Dijk, 2009; Wang & Davis, 2014; Smith, Timoshevskiy & Saraceno, 2020). This phenomenon of genetic material elimination was first discovered at the end of the 19th century by T. Boveri in parasitic nematodes, namely *Parascaris univalens* (reviewed in: Tobler, 1986; Kloc & Zagrodzinska, 2001; Grishanin *et al*., 2006; Wang *et al*., 2017). Using descriptive figures, Boveri depicted the process of genetic material removal in detail, showing that during the development of somatic cells, chromosomes become fragmented and that some regions are lost after the chromatids separate (reviewed in: Tobler, 1986; Müller & Tobler, 2000; Grishanin *et al*., 2006; Wang & Davis, 2014). This process was named ‘chromatin diminution’ by Herla (1893; cited in Tobler, 1986). Initially, Bovery considered such elimination to occur in all organisms and to be responsible for tissue differentiation (reviewed in: Tobler, 1986; Müller & Tobler, 2000; Grishanin *et al*., 2006). When this hypothesis was not confirmed in other animals, chromatin diminution was not subsequently investigated in detail. Nevertheless, DNA elimination and other processes in which genetic material is specifically removed have been found in various unrelated groups (Tobler, 1986; Dawley & Bogart, 1989; Kloc & Zagrodzinska, 2001; Burt & Trivers, 2009; Schön *et al*., 2009; Wang & Davis, 2014; Smith *et al*., 2020). Chromatin diminution and chromosome elimination were collectively named as ‘programmed DNA elimination’ (Wang & Davis, 2014). Herein, we use ‘programmed DNA elimination’ to refer to processes which were previously known as ‘chromatin diminution’.

Elimination of genetic material is involved in a number of processes across plants and animals, including elimination of B chromosomes, whole parental genome elimination, paternal genome elimination induced by parasitic elements, and selective genome elimination in plant and animal hybrids. Programmed elimination of genetic material may serve as a radical and irreversible form of genome competition, dosage compensation, epigenetic regulation, and inactivation of unnecessary or dangerous genetic elements.

Despite years of research into genetic material elimination, some major questions still remain unresolved: (*i*) the mechanisms of recognition of the sequences destined for elimination in different animals; (*ii*) whether these mechanisms are similar among organisms or are unique to each species; (*iii*) why elimination occurs only in some organisms while the vast majority are not able to eliminate DNA; (*iv*) whether there are predisposing factors for DNA elimination; and (*v*) whether it might be possible to use these mechanisms for the manipulation of the genome in research on selection, medicine, and agriculture.

In this review, we discuss the elimination of genetic material in different plants and animals, mechanisms of genetic material elimination, and possible functions of the elimination of DNA sequences. Although brief descriptions of different cases of programmed DNA elimination are available, with a specific focus on the known mechanisms, these mechanisms vary significantly and, thus, each case deserves detailed attention.

## II. UNSELECTIVE ELIMINATION OF GENETIC MATERIAL

Ionising radiation, cytotoxic agents, or deficiencies of some substances can lead to the spontaneous elimination of genetic material from the cell nucleus (Fenech & Crott, 2002; Lindberg *et al*., 2007; Fenech, 2010; Luzhna, Kathiria & Kovalchuk, 2013). Such stress factors can initiate double‐strand breaks, leading to the formation of acentric chromosome fragments that are unable to attach to the spindle during mitosis and remain in the cytoplasm (Fenech & Crott, 2002; Lindberg *et al*., 2007; Fenech, 2010; Luzhna *et al*., 2013). Moreover, merotelic kinetochore orientation, aberrations in chromosome condensation, cohesion, disjunction defects, and telomere–telomere fusion all can cause chromosomal loss even during normal cell division (Fenech *et al*., 2011; Gregan *et al*., 2011; Ganem & Pellman, 2012). After cell division, unselectively eliminated whole chromosomes or their fragments are usually enclosed in micronuclei and subsequently degraded (Crasta *et al*., 2012). Inactive X and Y chromosomes are frequently lost in ageing cells (Stone & Sandberg, 1995; Jones, York & Jackson‐Cook, 2012). Massive chromosomal loss has also been reported in cells undergoing oncological transformation (Gisselsson, 2008; Negrini, Gorgoulis & Halazonetis, 2010; Fenech *et al*., 2011). In addition to chromosomal lagging and the formation of chromosomal bridges, cancer cells are also characterised by the extrusion of genetic material or even whole chromosomes in micronuclei (Shimizu, Shimura & Tanaka, 2000; Utani, Okamoto & Shimizu, 2011; Kwon, Leibowitz & Lee, 2020). In cancer cells, micronuclei can persist even after several rounds of mitotic divisions and can even return to the main nucleus (Crasta *et al*., 2012; Zhang, Leibowitz & Pellman, 2013). Moreover, micronuclei can be a source of rearranged chromosomes during chromothripsis (Stephens *et al*., 2011; Crasta *et al*., 2012). Chromosomes enclosed in micronuclei undergo intensive fragmentation but segregate again with the rest of chromosomes during the next round of division (Crasta *et al*., 2012). In the nucleus, the damaged chromosome then undergoes non‐homologous end joining, resulting in highly rearranged chromosomal structures (Crasta *et al*., 2012; Zhang *et al*., 2013). Stress‐induced elimination of genetic material is non‐selective and does not occur at specific stage of ontogenesis.

## III. PROGRAMMED ELIMINATION OF GENETIC MATERIAL IN DIFFERENT ORGANISMS

**Fig 1:**
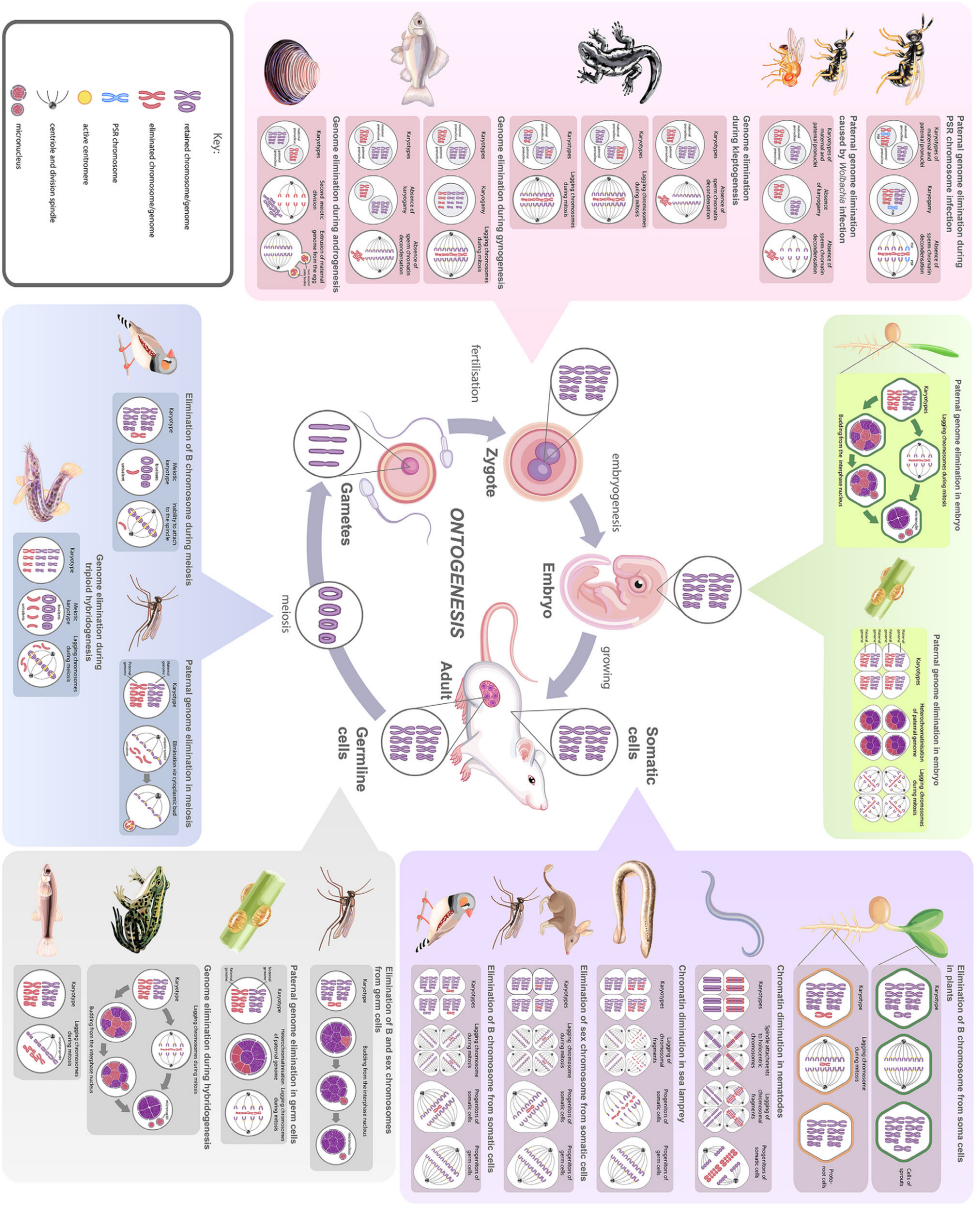
Overview of programmed DNA elimination during ontogenesis in multicellular model organisms. Normal ontogenesis without programmed DNA elimination is represented in the centre. Chromatin diminution, elimination of supernumerary and sex chromosomes, paternal genome elimination, parasitically induced genome elimination, and elimination of one parental genome in hybrids are shown according to the timing of elimination during ontogenesis. For specific details of each of the cases of programmed DNA elimination see Figs 2, 3, 4, 5, 6, 7, 8. GRC, germline‐restricted chromosome; PSR, paternal sex ratio.

Programmed elimination of genetic material has been found to be mediated by a variety of unrelated processes that appear independently across different taxa. In contrast to the unselective elimination of genetic material, programmed elimination of genetic material is highly specific for eliminating DNA sequences and usually occurs during specific stages of ontogenesis (see Fig. 1 for an overview).

### (1) Programmed DNA elimination

**Fig 2:**
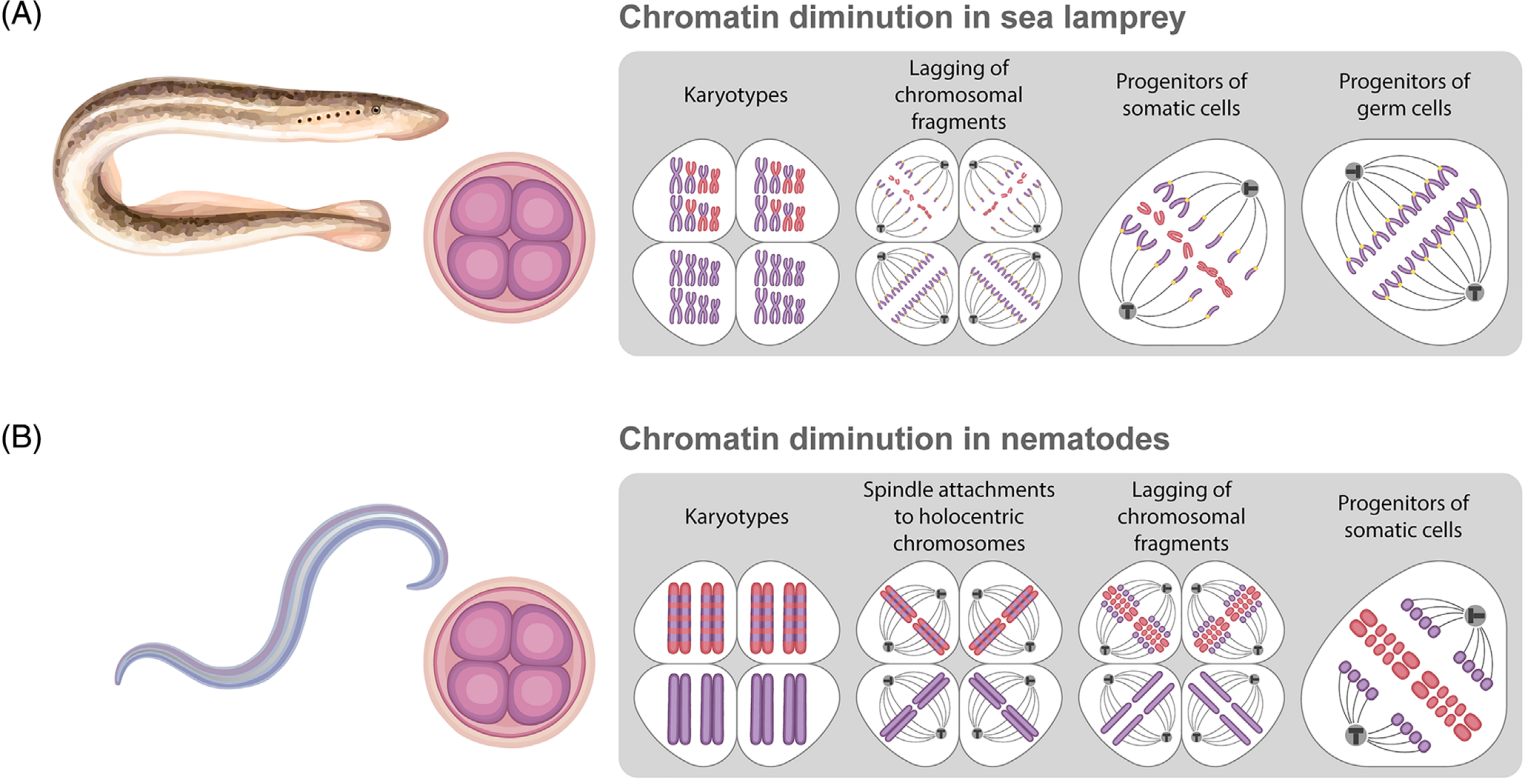
Chromatin diminution in sea lamprey (A) and parasitic nematode (B). See Fig. 1 for key. The image of the four‐cell embryo indicates that elimination takes place during early developmental stages. Eliminated (red) and retained (violet) chromosomes and their fragments are shown in the karyotype and during mitosis in the boxed images on the right. (A) In sea lamprey, the eliminated fragments or whole chromosomes do not attach to the spindle and lag during anaphase. Progenitors of germ cells keep their genome intact. (B) During chromatin diminution in progenitors of somatic cells in nematodes, the eliminated fragments of holocentric chromosomes do not attach to the spindle and are eliminated during anaphase. Progenitors of germ cells keep their genome intact. See text for further details.

Programmed DNA elimination collectively refers to elimination of chromosomal fragments from progenitors of somatic cells in different multicellular organisms and ciliate protozoa (Tobler, 1986; Kloc & Zagrodzinska, 2001; Grishanin *et al*., 2006; Wang & Davis, 2014; Smith *et al*., 2020) (Fig. 2; see online Supporting Information, Table S1). Programmed DNA elimination has been found in species from different classes of ciliates [Oligohymenophorea (*Paramecium* and *Tetrahymena*), Spirotrichea (*Euplotes*, *Oxytricha*, and *Stylonychia*)], and in at least 11 species of parasitic nematodes, 8 copepods, and in lampreys, and hagfish (Tobler, 1986; Nakai, Kubota & Kohno, 1991; Prescott, 1994; Grishanin *et al*., 2006; Smith *et al*., 2009; Wang & Davis, 2014). In multicellular organisms, programmed DNA elimination occurs in the progenitors of somatic cells during early developmental stages (usually at the 2–6 cleavage divisions stage, but in lampreys between the gastrula and blastula stages) (Fig. 2) (Tobler, 1986; Kloc & Zagrodzinska, 2001; Grishanin *et al*., 2006; Smith *et al*., 2020). In ciliates, programmed DNA elimination occurs during macronucleus formation and is accompanied by genome rearrangements (Tobler, 1986; Prescott, 1994; Mochizuki *et al*., 2002; Fang *et al*., 2012). Note that the macronucleus is functionally similar to the somatic cell nucleus in metazoans (Prescott, 1994). Genomes of germ cell progenitors in multicellular organisms and micronuclei in ciliates remain intact and retain all DNA sequences (Tobler, 1986; Kloc & Zagrodzinska, 2001; Grishanin *et al*., 2006; Smith *et al*., 2020).

During DNA elimination, chromosomes undergo radical and irreversible rearrangements; subtelomeric and/or interstitial DNA sequences are excised and subsequently degraded (Beermann, 1977; Tobler, Etter & Müller, 1992; Goday & Pimpinelli, 1993; Müller & Tobler, 2000; Clower *et al*., 2016; Grishanin & Zagoskin, 2018) (Fig. 2). In nematodes, following DNA breaks chromosomal fragment ends are healed with *de novo* telomere addition (Müller & Tobler, 2000; Wang *et al*., 2020) (Fig. 2B). In other species, chromosomal fragments may fuse to form new retained chromosomes (Beermann, 1977; Kubota *et al*., 2001; Grishanin & Zagoskin, 2018; Timoshevskiy, Timoshevskaya & Smith, 2019). Not only chromosomal fragments, but whole chromosomes can also be eliminated from progenitors of somatic cells during the early embryonic development of lampreys and hagfish (in lampreys, this process is also known as ‘programmed genome rearrangements’) (Nakai *et al*., 1991; Smith *et al*., 2009; Covelo‐Soto *et al*., 2014; Timoshevskiy *et al*., 2016, 2019). The proportion of eliminated genomes in different species usually varies from 25 to 90%; however, in some ciliate protozoa or copepod species, it can reach 98% of all genomic DNA (Tobler, 1986; Kloc & Zagrodzinska, 2001; Grishanin *et al*., 2006; Smith *et al*., 2009; Fang *et al*., 2012; Wang & Davis, 2014; Wang *et al*., 2017). Eliminated sequences usually include high‐copy tandem repeats, other repetitive sequences including copies of transposons, and unique sequences that are known to participate in gametogenesis but are not essential for somatic cells (Aeby *et al*., 1986; Kloc & Zagrodzinska, 2001; Grishanin *et al*., 2006; Fang *et al*., 2012; Wang & Davis, 2014; Smith *et al*., 2020) (Table S1).

Since the discovery of programmed DNA elimination, it has been considered relevant to cell differentiation mechanisms and the segregation of germ and somatic cell lines (Tobler, 1986; Smith *et al*., 2012; Wang & Davis, 2014; Wang *et al*., 2017; Timoshevskiy *et al*., 2019). In addition, programmed DNA elimination may affect gene expression and regulate the amount of heterochromatin in progenitors of somatic cells (Tobler, 1986; Kubota *et al*., 2001; Grishanin *et al*., 2006; Smith *et al*., 2012; Grishanin, 2014; Wang *et al*., 2017; Timoshevskiy *et al*., 2019) (Table S1). Moreover, increasing evidence suggests that approximately 5–10% of genes (depending on the species) are eliminated in nematodes (Wang *et al*., 2017). The majority of these eliminated genes are responsible for spermatogenesis, suggesting a particular role of programmed DNA elimination in regulation of the development and maturation of male germ cells (Wang *et al*., 2017). However, the most important function of DNA elimination appears to be the regulation of parasitic elements in the genomes of these organisms, since large parts of eliminated sequences are represented by transposons or their fragments (Aeby *et al*., 1986; Schoeberl & Mochizuki, 2011; Fang *et al*., 2012; Smith *et al*., 2012; Grishanin, 2014; Wang *et al*., 2017) (Table S1). One of the most fascinating examples bridging the emergence of programmed DNA elimination with transposable element control during oogenesis can be found in some copepod species (Sun *et al*., 2014). In these species, during oogenesis the size of the oocyte genome increases dramatically (in some species up to 100 times) due to uncontrolled activity of mobile elements. After fertilisation, DNA elimination purges these elements, restoring normal genome size.

### (2) Elimination of supernumerary chromosomes

**Fig 3:**
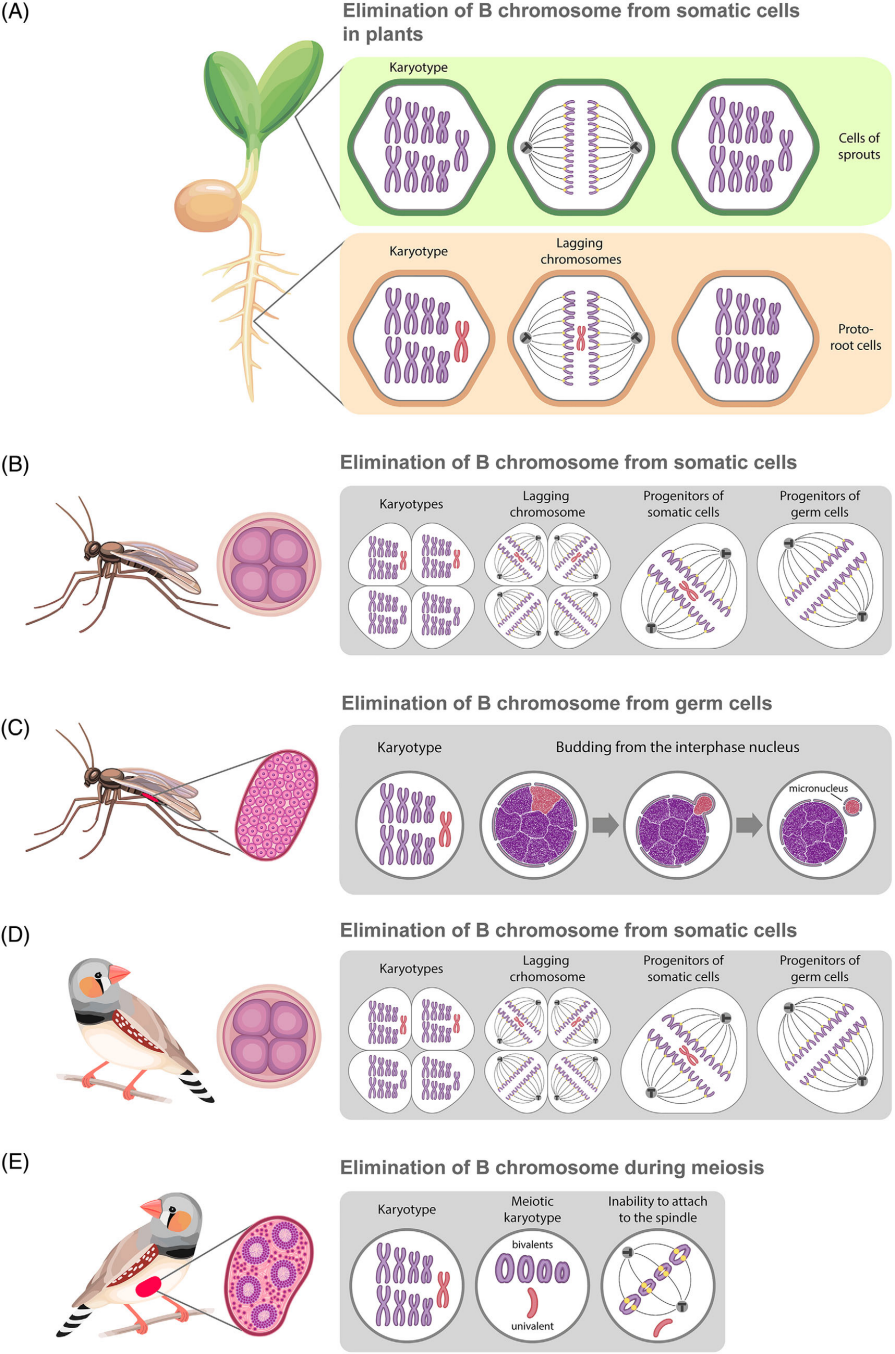
Supernumerary chromosome elimination in goatgrass (A), sciarid flies (B, C) and the zebra finch (D, E). Eliminated (red) and retained (violet) chromosomes are indicated in karyotypes in cells during interphase, in mitosis, and in meiosis in the boxed images on the right. The images of the four‐cell embryo or gonad indicate that elimination takes place during early developmental stages or during gametogenesis, respectively. (A) Elimination of the B chromosome occurs in proto‐root cells of the plant embryo but not in cells from the upper part of the plant. (B, D) Elimination of the B chromosome occurs only in progenitors of somatic cells of the embryo in sciarid flies and the zebra finch. (C) Elimination of the B chromosome *via* budding from the interphase nucleus occurs in germ cells of sciarid flies. (E) Elimination of the B chromosome during meiosis in zebra finch males. See text for further details.

Whole chromosomes can be eliminated from somatic cells, although they remain preserved in germ cells of at least one of the sexes (Gerbi, 1986; Hennig, 1986; Herrick & Seger, 1999; Torgasheva *et al*., 2019; Smith *et al*., 2020) (Fig. 3; Table S1). Individual chromosome elimination usually occurs during cell division and is not associated with chromosomal rearrangements (Gerbi, 1986; Herrick & Seger, 1999; Kloc & Zagrodzinska, 2001; Grishanin *et al*., 2006; Torgasheva *et al*., 2019; Smith *et al*., 2020) (Fig. 3). Such chromosomes are known as supernumerary or B chromosomes, and are usually not essential for survival (sometimes they are considered harmful) (Burt & Trivers, 2009; Jones, 2012; Houben *et al*., 2014). Nevertheless, B chromosomes undergo programmed elimination in selected organisms. When supernumerary chromosomes become highly specific to germ cells and are absent in somatic cells, they are frequently known as germline‐restricted or germline‐limited chromosomes (Gerbi, 1986; Nakai *et al*., 1991; Pigozzi & Solari, 1998; Goday & Esteban, 2001; Staiber, 2006; Smith *et al*., 2009; Torgasheva *et al*., 2019).

Germline‐restricted chromosomes (GRCs) have been discovered in insects and in some vertebrates, including songbirds (order Passeriformes) (Gerbi, 1986; Nakai *et al*., 1991; Pigozzi & Solari, 1998; Goday & Esteban, 2001; Staiber, 2006; Smith *et al*., 2009; Covelo‐Soto *et al*., 2014; Torgasheva *et al*., 2019) (Fig. 3; Table S1). Supernumerary chromosomes, or В chromosomes, found widely in plants and animals, tend to be maintained in germ cells that allows transmission to the next generation (Pigozzi & Solari, 1998; Goday & Esteban, 2001; Camacho, 2005; Burt & Trivers, 2009; Jones, 2012). However, they are frequently removed from somatic cells (Pigozzi & Solari, 1998; Goday & Esteban, 2001; Camacho, 2005; Burt & Trivers, 2009; Jones, 2012). Different individuals, even from the same population, may have different numbers of В chromosomes or lack them completely (Camacho, 2005; Jones, 2012). For example, in some plants (Jones & Rees, 1982), B chromosomes are found only in above‐ground parts whereas they are eliminated in root cells (Ruban *et al*., 2020) (Fig. 3A). GRCs and other B chromosomes are thought to have originated from autosomes or their fragments (Burt & Trivers, 2009; Jones, 2012; Houben *et al*., 2014). A recent study showed that GRCs in the fungus gnat *Sciara coprophila* resulted from an ancient introgression event possibly *via* interspecific hybridisation (Hodson *et al*., 2021). Although these chromosomes usually accumulate repetitive DNA organised into large heterochromatic blocks, they can contain protein‐coding or noncoding RNA genes that play key roles in gametogenesis and germ cell development (Tobler, 1986; Herrick & Seger, 1999; Houben *et al*., 2014; Biederman *et al*., 2018; Kinsella *et al*., 2019; Torgasheva *et al*., 2019; Malinovskaya *et al*., 2020; Hodson *et al*., 2021). Sequencing data showed that genes responsible for gametogenesis and embryonic development have been continuously added to GRCs during their evolution (Kinsella *et al*., 2019). Thus, the elimination of such chromosomes from somatic cells can affect germ cell and somatic cell segregation (Gerbi, 1986; Goday & Esteban, 2001) (Table S1). Despite originating as parasitic elements, these chromosomes possess mechanisms to prevent their elimination from germ cells, ensuring their survival and preferential transmission to the gametes (Gerbi, 1986; Goday & Esteban, 2001; Jones, 2012; Houben *et al*., 2014; Malinovskaya *et al*., 2020).

### (3) Sex chromosome elimination during dosage compensation and sexual differentiation

**Fig 4:**
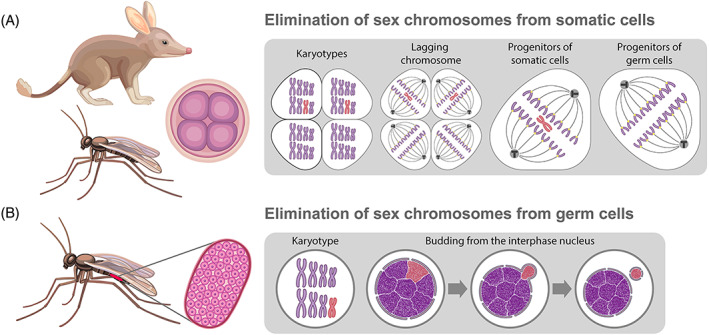
Sex chromosome elimination in bandicoots (A) and sciarid flies (A, B). Eliminated (red) and retained (violet) chromosomes are indicated in karyotypes in cells during interphase and in mitosis in the boxed images on the right. The images of the four‐cell embryo or gonad indicate that elimination takes place during early developmental stages or during gametogenesis, respectively. (A) Elimination of sex chromosomes only from progenitors of somatic cells occurs in the embryo in bandicoots and sciarid flies. (B) Elimination of sex chromosome *via* budding from the interphase nucleus in germ cells of sciarid flies. See text for further details.

Another relevant process is the elimination of whole chromosomes during sex determination and dosage compensation (Herrick & Seger, 1999; Kloc & Zagrodzinska, 2001; Burt & Trivers, 2009; Wang & Davis, 2014; Smith *et al*., 2020) (Fig. 4; Table S1). Usually, dosage compensation involves the selective inactivation of paternal (or less frequently, maternal) sex chromosomes (Herrick & Seger, 1999; Deakin *et al*., 2009). The elimination of inactivated sex chromosomes for irreversible and extreme dosage compensation has been reported in some invertebrates and in vertebrates, including marsupials (e.g. bandicoots from the orders Peramelidae and Peroryctidae, and pseudocheirid opossums) and at least two eutherian species, the spiny mouse (*Acomys* sp. from Tanzania) and Oregon meadow mouse (*Microtus oregoni*) (Hayman & Martin, 1965; Watson, Margan & Johnston, 1998; Johnston *et al*., 2002; Castiglia, Makundi & Corti, 2007; Smith *et al*., 2020) (Table S1). Notably, in marsupials, sex chromosomes can be eliminated only from certain somatic cells (Close, 1984). For example, the Y chromosome can be eliminated in somatic tissues in males and the X chromosome can be eliminated in somatic tissues in females (Watson *et al*., 1998).

In invertebrates, sex chromosome elimination is extremely variable, ranging from the elimination of one of the X chromosomes from somatic and germ cells in fungus gnats (*Sciara* spp., Sciaridae, Diptera) to several rounds of elimination of paternal sex chromosomes in the hessian fly *Mayetiola destructor* (Cecidomyiidae, Diptera) (Stuart & Hatchett, 1991; Sánchez & Perondini, 1999; Goday & Esteban, 2001; Burt & Trivers, 2009; Sánchez, 2014) (Fig. 4B; Table S1). In males, elimination of both X chromosomes may occur during early embryonic division, allowing sexual differentiation [for example, in springtails (Collembola)] (Dallai *et al*., 2001; Dallai, Fanciulli & Frati, 2004; Burt & Trivers, 2009) (Table S1). In the nematode genus *Strongyloides*, only certain parts of chromosomes are eliminated in a sex‐dependent manner (Streit *et al*., 2016).

### (4) Paternal genome elimination

**Fig 5:**
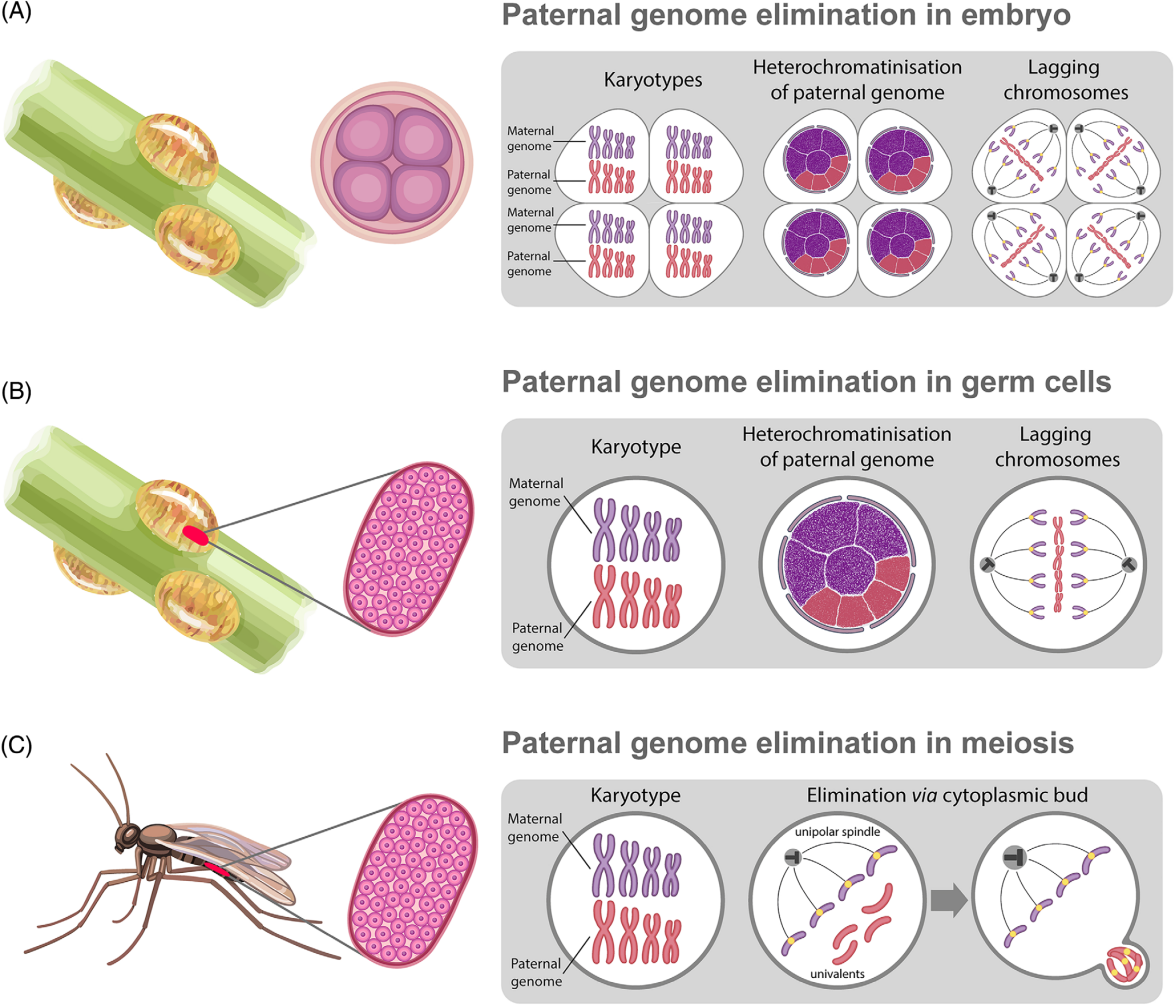
Paternal genome elimination in mealybugs (A, B) and sciarid flies (C). Eliminated (red) and retained (violet) chromosomes are indicated in karyotypes in cells during interphase and in mitosis in the boxed images on the right. The images of the four‐cell embryo or gonad indicates that elimination takes place during early developmental stages or during gametogenesis, respectively. (A) Elimination of all chromosomes from the paternal genome in all cells of the embryo during haplodiploid sex differentiation in mealybugs. (B) Elimination of all chromosomes from the paternal genome during germ cell development in mealybugs. (C) Elimination of all chromosomes from the paternal genome during monopolar spindle formation during meiosis in sciarid flies. See text for further details.

In certain invertebrates, not only sex chromosomes but the whole paternal genome can be eliminated during haplodiploid sex determination, characterised by the generation of haploid males and diploid females (Herrick & Seger, 1999; Burt & Trivers, 2009; Gardner & Ross, 2014; Sánchez, 2014; de la Filia, Bain & Ross, 2015) (Fig. 5; Table S1). Paternal genome elimination has been found in five arthropod orders, including mites (Phytoseiidae, Otopheidomenidae, and Ascoidea), flies (Sciaridae and Cecidomyiidae), springtails (Symphypleona), beetles (Cryphalini), and scale insects (Neococcoidea) (Gardner & Ross, 2014; de la Filia *et al*., 2015) (Table S1). In the haplodiploid sex‐determination system, males can develop from fertilised eggs and be diploid initially. However, during the early developmental stages, all paternal chromosomes are eliminated from somatic and germ cells (Herrick & Seger, 1999; Burt & Trivers, 2009; Gardner & Ross, 2014; Sánchez, 2014; de la Filia *et al*., 2015) (Fig. 5A; Table S1). In some organisms, paternal genome elimination does not involve somatic cells, but is restricted to gonia or meiotic cells (Herrick & Seger, 1999; Kloc & Zagrodzinska, 2001; Burt & Trivers, 2009; Gardner & Ross, 2014; de la Filia *et al*., 2015) (Fig. 5B, C). Despite the established role of paternal genome elimination in sex differentiation, researchers have hypothesised competition between the maternal and paternal genomes (Brown, 1965; Herrick & Seger, 1999) (Table S1). This hypothesis assumes the emergence of mutations that cause genome elimination in the maternal genome and protect the paternal genome from elimination (Herrick & Seger, 1999).

### (5) Induced genome elimination

**Fig 6:**
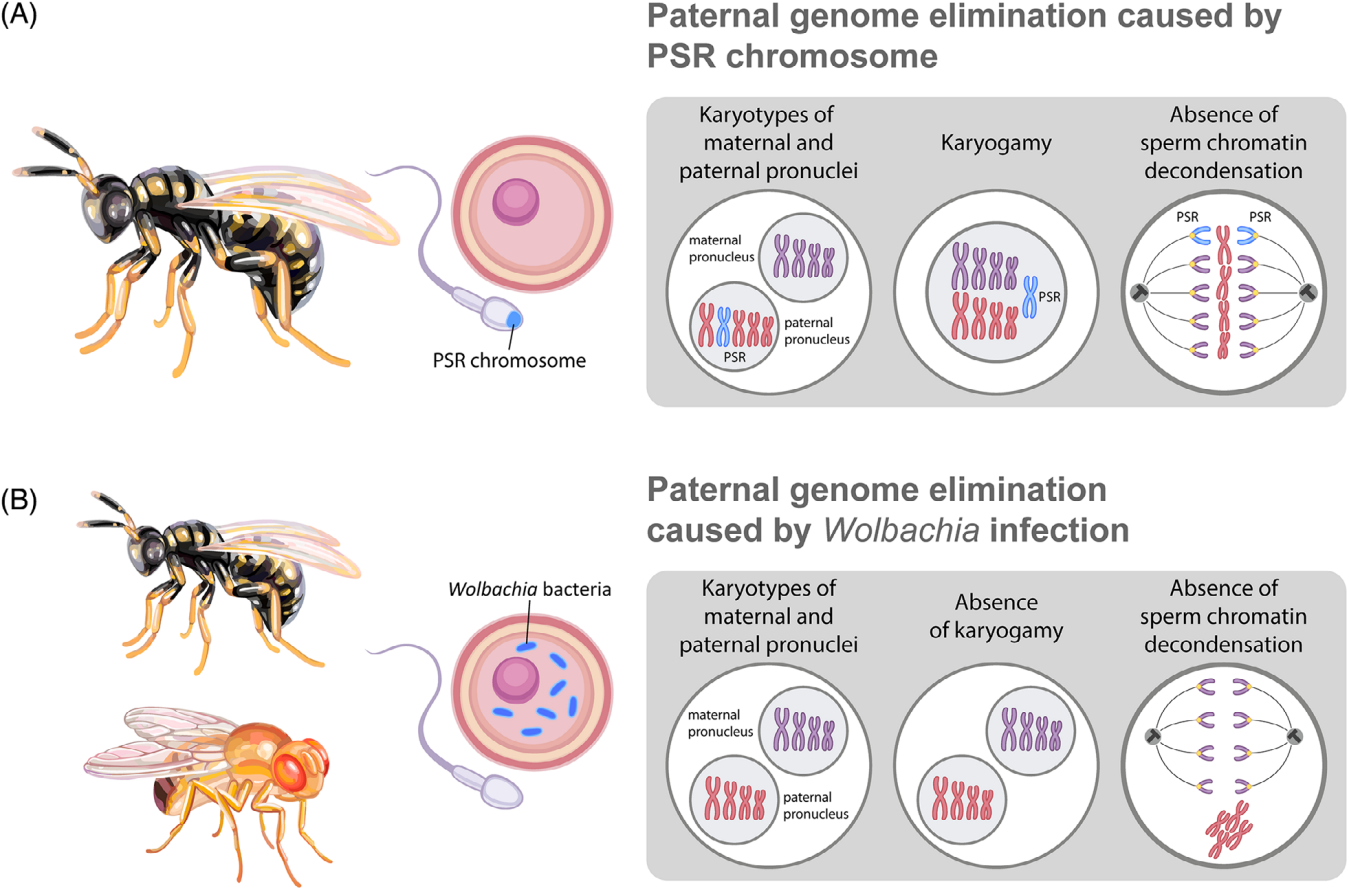
Paternal genome elimination caused by the paternal sex ratio (PSR) chromosome (A) and *Wolbachia* infection (B). Eliminated (red) and retained (violet) chromosomes are indicated in karyotypes in mitosis in boxed images on the right. The egg and sperm images indicate that elimination takes place after fertilisation. (A) Elimination of whole paternal genome after fertilisation during PSR chromosome infection in parasitoid wasp. The PSR chromosome is indicated in blue in the sperm chromatin and in the karyotypes. The PSR chromosome escapes the elimination of all other paternal chromosomes and segregates with the maternal chromosomes. (B) Elimination of whole paternal genome after fertilisation during *Wolbachia* infection (blue) in a fruit fly and a parasitoid wasp. The paternal pronucleus is unable to fuse with the maternal pronucleus; paternal chromatin remains condensed during the first zygotic division. See text for further details.

Selective elimination of one of the parental genomes has been found in organisms with a В chromosome called paternal sex ratio (PSR) or those infected with bacteria from the genus *Wolbachia* (Werren & Stouthamer, 2003; Werren, Baldo & Clark, 2008) (Fig. 6; Table S1). The PSR chromosome, found in parasitoid wasps (*Nasonia vitripennis*, *Trichogramma kaykai*), affects the sex ratio of the progeny (Nur *et al*., 1988; Stouthamer *et al*., 2001) (Fig. 6A; Table S1). Wasps have a haplodiploid sex determination system, in which diploid eggs develop into females and haploid eggs develop into males. Males transmit PSR chromosomes *via* the sperm (Werren, Nur & Eickbush, 1987; van Vugt *et al*., 2003). After fertilisation, the paternal genome is eliminated; however, the PSR chromosome avoids elimination and jumps to the female nucleus in the fertilised egg (van Vugt *et al*., 2003) (Fig. 6A; Table S1). Elimination of the paternal genome leads to the development of haploid embryos bearing the PSR chromosome (Reed & Werren, 1995; van Vugt *et al*., 2003; Swim, Kaeding & Ferree, 2012).

Other cases of induced genome elimination may occur during infection by the endosymbiotic bacterium *Wolbachia* (Werren *et al*., 2008). Although *Wolbachia* infects both males and females, it has various effects on host species (Herrick & Seger, 1999; Stouthamer *et al*., 2001; Werren *et al*., 2008). One of the most widespread effects of *Wolbachia* infection is cytoplasmic incompatibility, which involves paternal genome elimination (Werren *et al*., 2008). Cytoplasmic incompatibility occurs when a male infected with one *Wolbachia* strain is crossed with a female infected with another strain. After fertilisation, the paternal pronucleus is unable to fuse with the maternal pronucleus and is degraded in the cytoplasm, thus giving rise to haploid offspring (Fig. 6B; Table S1). Depending on the species, these offspring either die or develop into uninfected males (Reed & Werren, 1995; Tram, Ferree & Sullivan, 2003; Werren *et al*., 2008). Crosses between two parents infected with the same strain and between an infected female and an uninfected male do not lead to paternal pronucleus elimination *via* cytoplasmic incompatibility, allowing the normal development of infected diploid females (Reed & Werren, 1995; Herrick & Seger, 1999; Tram *et al*., 2003; Werren *et al*., 2008).

These cases of paternal genome elimination demonstrate how parasitic elements can effectively exploit the sex‐determination system of some insects to propagate to the progeny or decrease the number of uninfected individuals (Werren & Stouthamer, 2003; Werren *et al*., 2008; Burt & Trivers, 2009).

### (6) Chromosome elimination in interspecific plant hybrids

**Fig 7:**
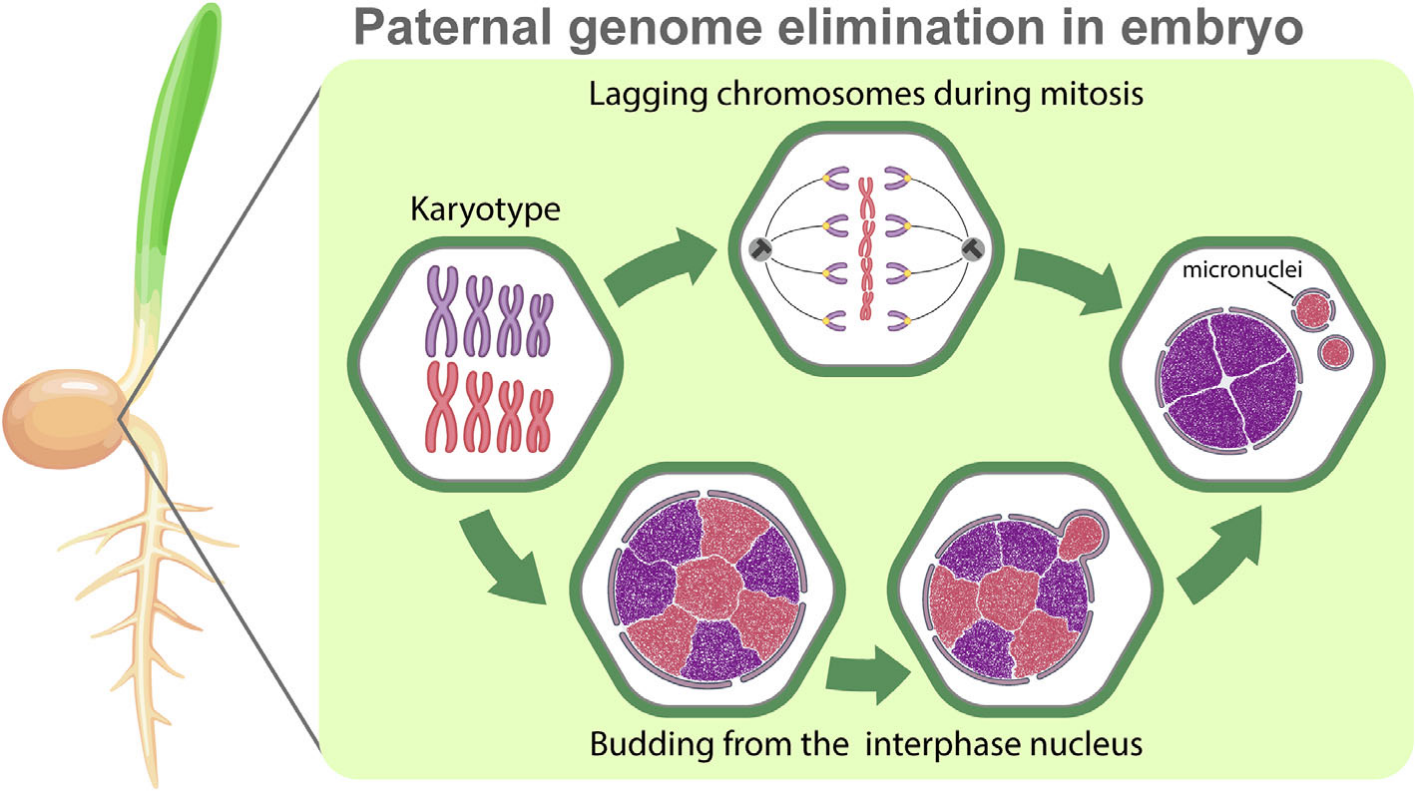
Elimination of one of the parental genomes in interspecific plant hybrids. Eliminated (red) and retained (violet) chromosomes are indicated in the karyotype in cells during interphase and mitosis in the boxed images on the right. Elimination of whole chromosomes from the genome of one parental species during early embryonic development in plant hybrids. Chromosomal elimination due to lagging during mitosis (upper row) and budding from the interphase nucleus (lower row). See text for further details.

Since early experiments involving artificial fusion of cells from different species (somatic cell hybrids), it was observed that chromosomes of one species can be lost after a series of divisions (Matsuya, Green & Basilico, 1968; Nabholz, Miggiano & Bodmer, 1969; Davidson, 1974). Elimination of chromosomes of one parental species also has been detected in some naturally occurring interspecific hybrids (Schwarzacher & Wachtler, 1983; Fujiwara *et al*., 1997; Mochida, Tsujimoto & Sasakuma, 2004; Sakai *et al*., 2007; Ishii *et al*., 2010) (Fig. 7). In both animals and plants, interspecific hybrids usually die (Coyne & Orr, 1998; Maheshwari & Barbash, 2011). Nevertheless, in the case of plants, such hybrids can be artificially rescued (Kasha & Reinbergs, 1976; Shimizu *et al*., 1999; Sakai *et al*., 2007; Houben, Sanei & Pickering, 2011; Yoshikawa *et al*., 2018). After fertilisation followed by elimination of one parental genome, the plant hybrid embryos must be placed in an artificial growth medium. Later, these embryos can be transplanted to soil where they develop into normal haploid plants but with aberrant meiosis. Therefore, plant breeders use spindle inhibitors (such as colchicine) to induce duplication of the haploid chromosomal set and the formation of diploid organisms (Kasha & Reinbergs, 1976; Forster & Thomas, 2005; Houben *et al*., 2011).

Elimination of one of the parental genomes occurs during early development and includes gradual chromosomal loss due to lagging in anaphase or the formation of chromosomal bridges (Bennett, Finch & Barclay, 1976; Gernand *et al*., 2005, 2006; Ishii *et al*., 2010) (Fig. 7; Table S1). Chromosomes or their fragments may also be eliminated through the budding of micronuclei from the interphase nuclei (Gernand *et al*., 2005, 2006) (Fig. 7; Table S1).

Selective elimination of one of the parental genomes in interspecific plant hybrids can be considered an example of a postzygotic barrier (Subrahmanyam & Kasha, 1973; Chan, 2011). In this case, only one genome can operate while the other fails to work normally due to cell cycle asynchronisation or centromere dysfunction, and is thus eliminated during early development (Subrahmanyam & Kasha, 1973; Chan, 2011).

### (7) Chromosome elimination during clonal and hemiclonal reproduction of animal interspecific hybrids

**Fig 8:**
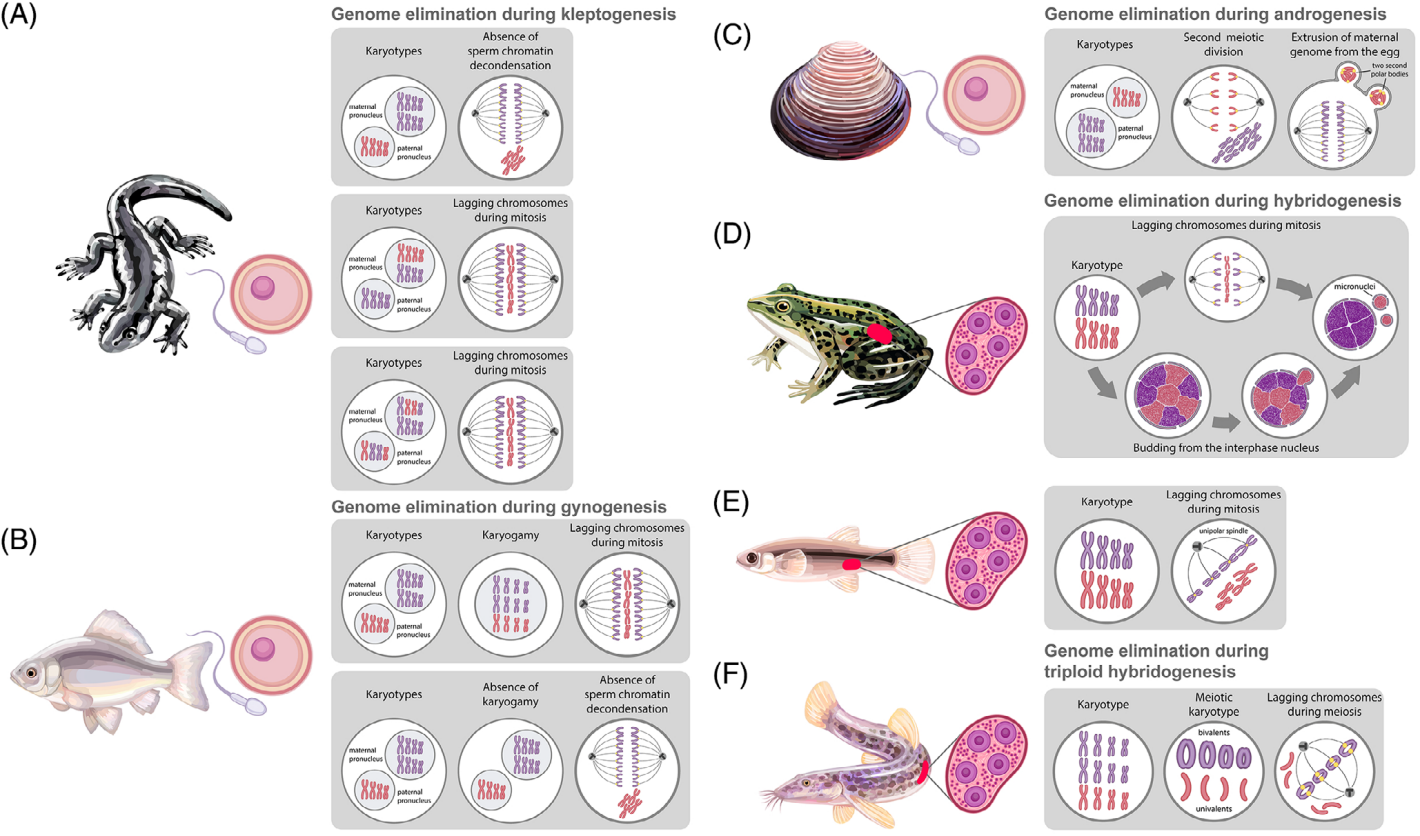
Elimination of one of the parental genomes in animal hybrids reproducing clonally *via* kleptogenesis (A), gynogenesis (B), androgenesis (C), hybridogenesis (D, E), and meiotic (triploid) hybridogenesis (F). Eliminated (red) and retained (violet) chromosomes are indicated in karyotypes and meiosis. The egg and sperm images indicate that elimination takes place after fertilisation; the gonad image indicates that elimination takes place during gametogenesis. (A) Elimination of the paternal (upper panel) or maternal (middle panel) genomes, or partial replacement of maternal chromosomes (lower panel) during kleptogenetic reproduction in hybrid salamanders from the genus *Ambystoma*. (B) Elimination of the paternal pronucleus after fusion with the maternal pronucleus (upper panel) and without fusion (lower panel) during gynogenetic reproduction in hybrid fishes from the genus *Carassius*. (C) Elimination of the maternal genome after fertilisation *via* the formation of two secondary polar bodies during androgenetic reproduction in hybrid molluscs from the genus *Corbicula*. (D, E) Mechanisms of parental genome elimination in hybrid water frogs from the genus *Pelophylax* (D) and poecilid fishes (E). (D) Chromosomes of one of the parental genomes are gradually lost *via* lagging during mitosis (upper row) or budding from the interphase nucleus (lower row). (E) Chromosomes of one of the parental species attach to the spindle while those of the other parental species are not capable of doing so. (F) Genome elimination during meiosis in triploid hybrid loach from the genus *Misgurnus*. Chromosomes from the double‐copy genome form bivalents that are able to attach to the spindle while those from the single‐copy genome form univalents that are unable to attach to the spindle, and hence are eliminated during anaphase. See text for further details.

Selective genome elimination has been observed in interspecific animal hybrids with clonal or hemiclonal reproductive modes (Dawley & Bogart, 1989; Schön *et al*., 2009; Stenberg & Saura, 2013; Schwander & Oldroyd, 2016; Stöck *et al*., 2021). One of the parental genomes or some of their parts may be eliminated in the interspecific hybrid during gametogenesis (hybridogenesis) or just after fertilisation (gynogenesis, kleptogenesis, and androgenesis) (Dawley & Bogart, 1989; Schön *et al*., 2009; Schwander & Oldroyd, 2016; Stöck *et al*., 2021) (Fig. 8; Table S1). In different hybrid forms, elimination of one of the parental genomes may occur immediately after fertilisation (all gynogenetic fish taxa; mole salamanders from the genus *Ambystoma*; androgenetic molluscs from the genus *Corbicula*, and stick insects from the genus *Bacillus*; gonochoric reduction in triploid carp *Carassius gibelio*), during mitotic division of germ cells (frogs *Pelophylax esculentus*, toads *Bufo baturae*, fish *Squalius alburnoides*, *Poeciliopsis monachal‐lucida*, and *Bacillus* stick insects), or directly during meiosis (loaches *Misgurnus anguillicaudatus*) (Cimino, 1972*a*; Tunner & Heppich, 1981; Komaru, Kawagishi & Konishi, 1998; Scali *et al*., 2003; Saitoh, Kim & Lee, 2004; Bogart *et al*., 2007; Morishima, Yoshikawa & Arai, 2008; Stöck *et al*., 2012; Zhang *et al*., 2015; Dedukh *et al*., 2020) (Fig. 8; Table S1).

Paternal genome, one of the maternal genomes or only some of their parts, are eliminated during kleptogenesis (Bogart *et al*., 2007). Kleptogenesis is a very old and successful reproductive strategy observed in more than 20 diploid, triploid, and tetraploid hybrids of North American mole salamanders from the genus *Ambystoma* (Bogart *et al*., 2007, 2009; Bi & Bogart, 2010; Bogart, 2019). Kleptogenesis includes some still unknown events that result in partial or complete replacement of one of the egg genomes with the sperm genome (Bogart, Elinson & Licht, 1989; Elinson *et al*., 1992; Bogart *et al*., 2007; Bogart, 2019) (Fig. 8A; Table S1).

Among asexual vertebrate organisms, gynogenesis is one of the most widespread reproductive modes (Dawley & Bogart, 1989; Schön *et al*., 2009; Stöck *et al*., 2021). It relies on the formation of unreduced gametes that are activated by sperm, but do not incorporate sperm genetic material (Dawley & Bogart, 1989; Schön *et al*., 2009; Neaves & Baumann, 2011; Stenberg & Saura, 2013; Stöck *et al*., 2021). The sperm pronucleus is usually unable to merge with the female pronucleus and subsequently degrades (Saat, 1991; Neaves & Baumann, 2011; Zhao *et al*., 2011; Stenberg & Saura, 2013; Zhang *et al*., 2015) (Fig. 8B; Table S1).

Natural androgenesis is known only in some cypress species (*Cypresses*), bivalves from the genus *Corbicula*, and occasionally in some fish species and *Bacillus* stick insects (Mantovani & Scali, 1992; Tinti & Scali, 1995; Komaru *et al*., 1998; Komaru, Ookubo & Kiyomoto, 2000; Ishibashi *et al*., 2003; Wang *et al*., 2011*b*; Morgado‐Santos *et al*., 2017). During androgenesis, the maternal genome is eliminated while the paternal genome [or genomes in the case of polyspermy (Mantovani & Scali, 1992; Tinti & Scali, 1995)] continues further development (Komaru *et al*., 1998, 2000; Ishibashi *et al*., 2003) (Fig. 8C; Table S1).

Hybridogenetic reproduction includes complete elimination of one parental genome in germline cells, thereby preventing chromosomal conflict during meiosis. Hybridity is restored after crossing such hybrids with complementary parental species (Dawley & Bogart, 1989; Schön *et al*., 2009; Schmidt *et al*., 2011; Kimura‐Kawaguchi *et al*., 2014; Stöck *et al*., 2021). Thus, hybridogenetic animals are also called ‘gamete parasites’, since they can reproduce with one of the parental species (Schultz, 1969; Tunner, 1973; Mantovani & Scali, 1992; Vrijenhoek, 1994; Schmidt *et al*., 2011; Unmack *et al*., 2019; Majtánová *et al*., 2021). Hybridogenesis has been observed in diploid hybrid fish including livebearers from the genus *Poeciliopsis* (Schultz, 1967, 1969), carp gudgeons (*Hypseleotris*) (Schmidt *et al*., 2011; Majtánová *et al*., 2021), chub (*Squalius*) (Carmona *et al*., 1997) and greenlings (*Hexagrammos*) (Kimura‐Kawaguchi *et al*., 2014; Munehara *et al*., 2016), *Bacillus* stick insects (Scali *et al*., 2003), water frogs (*Pelophyla*x) (Tunner, 1973), and triploid hybrid (known as triploid or meiotic hybridogenesis) loaches (*Misgurnus*) (Morishima *et al*., 2008), spined loaches (*Cobitis*) (Saitoh *et al*., 2004), chub (*Squalius*) (Alves, Coelho & Collares‐Pereira, 2001; Nabais *et al*., 2012), green toads (*Bufotes*) (Stöck *et al*., 2012) and water frogs (*Pelophylax*) (Dawley & Bogart, 1989; Vinogradov *et al*., 1990; Christiansen & Reyer, 2009; Dedukh *et al*., 2015) (Fig. 8D–F; Table S1).

Selective genome elimination in these organisms allows them to overcome hybrid sterility issues, specifically avoiding the mispairing of chromosomes during meiosis (Dawley & Bogart, 1989; Schön *et al*., 2009). For some hybrids, genome elimination prevents the acceleration of ploidy level, thus providing normal development after fertilisation (Dawley & Bogart, 1989; Elinson *et al*., 1992; Schön *et al*., 2009; Schwander & Oldroyd, 2016).

## IV. MECHANISMS OF PROGRAMMED ELIMINATION OF GENETIC MATERIAL

Elimination of genetic material may occur *via* various mechanisms (Table S1). Organisms can selectively exclude chromosomal fragments, whole chromosomes, or even entire parental genome. Moreover, eliminated sequences can include tandem repeats, mobile elements, and genes (Table S1). The genetic material destined for elimination needs to be recognised and removed appropriately from the nucleus. Elimination is typically accompanied by the epigenetic labelling of selected chromatin, which is especially true in ciliates where epigenetic markers have demonstrated an important role in elimination. In other organisms, the particular role of epigenetic modifications in labelling genomic sequences that should be retained or eliminated remains unknown.

### (1) Role of noncoding RNAs in programmed DNA elimination in ciliates

During programmed DNA elimination and rearrangements in ciliates, chromosomes undergo radical reorganisation, including their fragmentation, amplification and the removal of specific genetic sequences (Tobler, 1986; Grishanin *et al*., 2006; Mochizuki, 2010; Wang & Davis, 2014; Smith *et al*., 2020). During macronucleus formation, both chromosomal ends and internal eliminated sequences are removed. The remaining chromosomal fragments are re‐ligated to form new chromosomes followed by the addition of new telomeres. Such chromosomes undergo several rounds of endoreplication (Mochizuki *et al*., 2002; Mochizuki & Gorovsky, 2004; Mochizuki, 2010; Fang *et al*., 2012). Mechanisms involved in the recognition of eliminated or retained DNA sequences are known only for two classes of ciliates: Oligohymenophorea (*Paramecium* and *Tetrahymena*) and Spirotrichea (*Euplotes*, *Oxytricha*, and *Stylonychia*) (Mochizuki *et al*., 2002; Mochizuki & Gorovsky, 2004; Mochizuki, 2010; Fang *et al*., 2012; Nekrasova & Potekhin, 2018).

Short noncoding RNAs play a key role in the recognition of specific sequences; however, they act in a completely different way in Oligohymenophorea and Spirotrichea ciliates (Mochizuki & Gorovsky, 2004; Mochizuki, 2010; Fang *et al*., 2012). In Oligohymenophorea (*Paramecium* and *Tetrahymena*), short noncoding RNAs (also known as scanning RNAs or scnRNAs) recognise DNA sequences that should be eliminated from the chromosomes (Mochizuki *et al*., 2002; Lepère *et al*., 2008) (Table S1). scnRNAs are initially transcribed in newly formed micronuclei as double‐stranded RNA transcripts that are processed by the Dicer homolog (Mochizuki & Gorovsky, 2004; Malone *et al*., 2005; Lepère *et al*., 2009). These scnRNAs are loaded onto Argonaute and move to the old macronucleus (Noto *et al*., 2010). In the old macronucleus, the scnRNA pool becomes saturated by scnRNAs that complement the eliminated sequences in the genome (Mochizuki, 2010). After the saturation stage, scnRNAs move to new macronuclei where they mark the homologous sequences and attract histone methyltransferases, causing heterochromatin formation in these regions *via* H3K9me3 and H3K27me3 marks (Liu, Mochizuki & Gorovsky, 2004; Liu *et al*., 2007; Mochizuki, 2010) (Table S1). Finally, piggyBac transposase‐related proteins recognise the heterochromatin regions and cut them out (Baudry *et al*., 2009; Cheng *et al*., 2010).

In contrast to their role in Oligohymenophorea ciliates, short noncoding RNAs distinguish the retained sequences in Spirotrich ciliates (Fang *et al*., 2012). Moreover, in *Euplotes crassus*, *Oxytricha trifallax*, and *Stylonychia lemnae*, recognition is implemented by another type of short noncoding RNAs, namely Piwi‐interacting RNAs (piRNAs) which interact with Piwi proteins (Fang *et al*., 2012; Yerlici & Landweber, 2014) (Table S1). The piRNA subclass is abundant in the animal germline and is responsible for retrotransposon silencing (Carmell *et al*., 2007; Brennecke *et al*., 2008). In *E. crassus*, *O. trifallax*, and *S. lemnae*, piRNA precursors are formed in the old macronucleus during conjugation (Fang *et al*., 2012). After processing, piRNAs move to the new macronucleus and mark the sequences of protein‐coding and RNA genes that should be retained; the unmarked sequences become methylated on cytosine residues (Fang *et al*., 2012; Bracht, 2014; Yerlici & Landweber, 2014). Finally, the unmarked sequences are cut off using numerous domesticated transposases (Nowacki *et al*., 2009; Fang *et al*., 2012; Yerlici & Landweber, 2014) (Table S1). After RNA‐mediated elimination of DNA sequences in the developing macronucleus, the remaining fragments undergo massive rearrangements including inversions and translocations to produce functional genes in a process known as unscrambling (Prescott, 1999; Chen *et al*., 2014). Moreover, long‐noncoding RNAs derived from the paternal macronucleus serve as a template for guiding DNA unscrambling (Nowacki *et al*., 2009).

### (2) Cellular and molecular processes accompanying programmed DNA elimination in multicellular organisms

In other organisms with programmed DNA elimination, including nematodes, the relationship between noncoding RNAs and recognition of eliminated sequences has not yet been established. In nematodes, detailed transcriptome analysis of germ and somatic cells allowed the description of different noncoding RNA classes; but their role in programmed DNA elimination remains unknown (Wang *et al*., 2011*a*). Cellular processes accompanying programmed DNA elimination have been described in other species. In copepod (Beermann, 1977; Rasch & Wyngaard, 2008; Clower *et al*., 2016), nematode (Tobler, 1986; Tobler *et al*., 1992; Müller & Tobler, 2000), and lamprey (Timoshevskiy *et al*., 2016, 2019) species, DNA elimination can be observed morphologically during mitosis when eliminated chromatin remains in the cell equator after chromosomes have segregated to daughter cells (Fig. 2A, B; Table S1). However, DNA recognition and cutting are thought to occur during the interphase which precedes eliminating mitosis (Tobler, 1986; Magnenat, Tobler & Müller, 1999; Akifyev & Grishanin, 2005; Wang *et al*., 2020).

In nematodes, eliminated regions exhibit abnormal chromatin condensation and have more accessible chromatin structure but chromatin is not enriched by histone modifications, such as H3K4me3, H3K36me3, H4K20me1, H3K27me3, H3K9me2 and H3K9me3 (Goday *et al*., 1992; Niedermaier & Moritz, 2000; Wang *et al*., 2017) (Table S1). Moreover, in nematodes, eliminated regions of their holocentric chromosomes do not accumulate centromeric CENP‐A histone and do not form kinetochores, which leads to their inability to attach to the mitotic spindle (Kang *et al*., 2016) (Table S1). Analysis of genomes in germline and somatic cells in different parasitic nematodes with programmed DNA elimination showed that regions of chromosomal breaks are not well conserved among different species and depend strongly on species divergence (Bachmann‐Waldmann *et al*., 2004; Wang *et al*., 2017). Moreover, chromosomal breakage does not depend on sequence motifs or structure of specific sequences (Wang *et al*., 2017). Such chromosomal breaks occur randomly within 3–6 kb regions and healing of the breaks occurs by telomere addition (Wang *et al*., 2017). After elimination, fragments remain in the cytoplasm, are enclosed into micronuclei and degrade while the other chromosomes are successfully separated (Goday *et al*., 1992; Niedermaier & Moritz, 2000; Wang *et al*., 2020).

In the genus *Cyclops* (Copepoda), the removal of chromosomal fragments including interstitial ones does not increase chromosomal number (Beermann, 1977; Grishanin & Akif'ev, 1993). Eliminated DNA forms circular structures enclosed in granules where it is subsequently degraded (Beermann & Meyer, 1980; Grishanin & Akif'ev, 1993; Grishanin & Zagoskin, 2018). Based on these observations, researchers have suggested a model of DNA elimination in *Cyclops* species (Beermann & Meyer, 1980). DNA sequences destined for elimination are looped out of the chromosomes, excited and then ligated to form a circle, while the remaining chromosomal fragments re‐join together (Beermann & Meyer, 1980; Grishanin & Akif'ev, 1993; Grishanin & Zagoskin, 2018). Interestingly, in lampreys, heterochromatin modifications (5meC, H3K9me3) accumulate in the eliminated regions of chromosomes and in micronuclei comprised of eliminated chromatin (Timoshevskiy *et al*., 2016) (Table S1). The eliminated chromatin is enclosed in micronuclei, and subsequently degraded in the cytoplasm (Timoshevskiy *et al*., 2016).

### (3) B or sex chromosomes are eliminated through chromosomal lagging

Whole chromosome elimination is usually mediated by centromere malfunction, causing inability to attach to the spindle and lag during anaphase of mitosis or meiosis (Goday & Esteban, 2001; Burt & Trivers, 2009; Schoenmakers *et al*., 2010; Escribá, Giardini & Goday, 2011; Jones, 2012; Staiber, 2012, 2014). In *Aegilops speltoides* (goatgrass), B chromosomes are eliminated from roots *via* nondisjunction during mitosis and lag during anaphase (Ruban *et al*., 2020). However, in some species, the elimination of chromosomes is characterised by additional epigenetic modifications during certain stages of development (Goday & Esteban, 2001; Schoenmakers *et al*., 2010; Escribá *et al*., 2011; Staiber, 2012).

The germline restricted chromosome (GRC), found in songbirds, is one of the best studied examples of elimination of supernumerary chromosomes (Fig. 3D, E; Table S1). Before elimination in both zebra finch (*Taeniopygia guttata*) and Bengalese finch (*Lonchura domestica*) males, GRC accumulates histone modifications, such as H3K9me2 and H3K9me3 and, at least in the zebra finch, GRC becomes hypermethylated on histone H4K20 during early meiotic prophase (Goday & Pigozzi, 2010; Schoenmakers *et al*., 2010; Del Priore & Pigozzi, 2014). In meiotic prophase, the GRC showed a decreased number of RAD51 (radiation‐repair protein 51) and γH2AX (phosphorylated on serine 139 histone H2AX) foci as well as weaker TUNEL (terminal deoxynucleotidyl transferase dUTP nick end labelling) staining compared to other chromosomes, suggesting a low level of double‐strand break formation on this chromosome. At leptotene and pachytene, GRC becomes intensively acetylated on histone H4K16 and shows strong association with SUMO (small ubiquitin‐related modifier‐1) and HP1 (heterochromatin protein 1) proteins. These epigenetic marks maintain until metaphase I. In the late prophase and metaphase I, GRC accumulates hypophosphorylated histone Н3S10 and ubiquitylated histone H2AK119 and exhibits failure of loading of INCENP (inner centromeric protein). It was suggested that these epigenetic modifications can cause kinetochore and/or centromere malfunction (Goday & Pigozzi, 2010; Schoenmakers *et al*., 2010; Del Priore & Pigozzi, 2014) (Table S1). Histone phosphorylation at H3S10 and H3S28 during mitosis and meiosis is closely linked to chromosome condensation and is mediated by Aurora B kinase (Giet & Glover, 2001; Goto *et al*., 2002). It appears that the GRC cannot attach to the microtubules of the spindle during metaphase of the first meiotic division (Schoenmakers *et al*., 2010; Del Priore & Pigozzi, 2014). After elimination, the GRC forms a micronucleus, which is actively stained by TUNEL, suggesting intensive DNA fragmentation followed by degradation (Pigozzi & Solari, 1998; Goday & Pigozzi, 2010; Schoenmakers *et al*., 2010; Del Priore & Pigozzi, 2014) (Table S1).

Morphologically distinct condensation has been observed during chromosome elimination from somatic cells in *Sciara* fungus gnats (Sciaridae, Diptera) and in the midge *Acricotopus lucidus* (Cecidomyiidae, Diptera) (Perondini & Ribeiro, 1997; Staiber, 2006) (Table S1). In *Sciara* fungus gnats, phosphorylation of H3S10 in chromosomes occurs normally during mitotic prophase; however, eliminating chromosomes show abnormalities in H3S10 dephosphorylation during late metaphase (Escribá & Goday, 2013). In normal cells, dephosphorylation of H3S10 and H3S28 accompanies sister chromatid separation and causes Aurora B dissociation from metaphase chromosomes (Adams *et al*., 2001; Goto *et al*., 2002) (Table S1). Thus, H3S10 and H3S28 dephosphorylation only takes place in the retained chromosomes, but not in the eliminated ones, leading to their proper segregation in contrast to eliminated chromosomes (Escribá & Goday, 2013) (Figs 3B, 4A). Aberrant chromatid separation has also been reported for the eliminated additional chromosomes in *A. lucidus* (Cecidomyiidae) (Staiber, 2006) (Table S1).

In *Sciara* fungus gnats, elimination of supernumerary and paternal sex chromosomes occurs not only *via* lagging during cell divisions in somatic cells but also *via* micronucleus budding in germ cells (Goday & Esteban, 2001) (Figs 3C, 4B; Table S1). In germ cells, one of the two paternal X chromosomes and some L chromosomes are semicondensed and hence, are morphologically visible, resembling prometaphase chromosomes; however, the other chromosomes are fully decondensed. During elimination, one of the semicondensed chromosomes is connected to the nuclear membrane (presumably *via* the lamin B receptor) and extruded from the nucleus to the cytoplasm *via* bud formation (Perondini & Ribeiro, 1997).

Sex chromosome inactivation and heterochromatinisation is known in many species including marsupials. However, in some marsupials, one of the sex chromosomes (usually the paternal chromosome) is eliminated from the somatic cells (reviewed in: Deakin *et al*., 2009; Smith *et al*., 2020) (Fig. 4A; Table S1). Notably, Х chromosome inactivation in all marsupials is not controlled by the long noncoding RNA XIST (X‐inactive specific transcript), unlike in placental mammals (Deakin *et al*., 2009). Moreover, histone modifications typical for heterochromatin, such as H3K27me3 and Н3K9me2, have not been detected in the inactive X chromosome in somatic cells of marsupials (Kohlmaier *et al*., 2004; Deakin *et al*., 2009). Nevertheless, differences in DNA methylation levels were seen in marsupial X chromosomes (Waters *et al*., 2018). To explain the mechanisms causing elimination of sex chromosomes, a replication delay of the inactive X chromosomes (Johnston *et al*., 2002) or mitotic errors (Close, 1984) have been suggested.

### (4) Inability to form chromosomes during metaphase of mitotic or meiotic division leads to the elimination of paternal chromatin

In various species, paternal genome elimination can occur at different ontogenetic stages. Paternal genome elimination has been observed during early embryonic development from progenitors of germ cells, during germ cell divisions, as well as during meiosis (Herrick & Seger, 1999; Burt & Trivers, 2009; Gardner & Ross, 2014; de la Filia *et al*., 2015) (Fig. 5A, B). In scale insects (Coccidae, Hemiptera), preliminary heterochromatinisation of the paternal genome precedes its elimination (Brown & Nur, 1964; Nur, 1990; Ross, Pen & Shuker, 2010; Prantera & Bongiorni, 2012). In these animals, paternal chromosomes can be heterochromatinised early in development and eliminated from germ cell genomes during spermatogenesis (e.g. lecanoid and *Comstockiella* scale insects), or they may be eliminated during early development in males (diaspidid scale insects) (Brown & Nur, 1964; Nur, 1990; Ross *et al*., 2010) (Fig. 5A, B; Table S1). In male embryos of species with embryonic (diaspidid) elimination, paternal chromatids fail to disjoin during early‐cleavage divisions (Herrick & Seger, 1999) (Table S1). Paternal chromosomes are eliminated due to lagging during anaphase (Fig. 5A). If a paternal chromosome escapes elimination during mitosis, it remains condensed until the next one, when it is eliminated (Brown, 1965). In lecanoid scale insects, before elimination, paternal chromosomes accumulate epigenetic markers typical of heterochromatin, such as H3K9me3, H4K20me3, and HP1‐related proteins (Bongiorni *et al*., 2007; Prantera & Bongiorni, 2012). During inverted meiosis in males of the citrus mealybug *Planococcus citri* (Coccidae, Hemiptera), both maternal and paternal chromosomes are sorted non‐randomly through a monopolar spindle (Bongiorni *et al*., 2004). As a result, spermatids with only maternally derived euchromatic chromosomes continue to develop while those with paternally derived heterochromatic chromosomes degenerate (Bongiorni *et al*., 2004).

Accumulation of epigenetic modifications in maternal and paternal genomes has been observed in *Liposcelis* booklice (Psocodea) and *Sciara* fungus gnats (Sciaridae, Diptera) (Goday & Ruiz, 2002; Greciano & Goday, 2006; Hodson *et al*., 2017) (Table S1). In *Sciara* fungus gnats, during early germ cell development, paternal chromosomes are known to accumulate H3K9ac, H3K14ac, H4K8ac, and H4K12ac, whereas maternally derived chromosomes exhibit the accumulation of H3K4me2 and H3K4me3. However, during gonial cell multiplication, the opposite epigenetic markers are seen (Goday & Ruiz, 2002; Greciano & Goday, 2006). Such epigenetic states of paternal chromosomes are maintained until meiosis, when the paternal chromosomes are eliminated (Esteban *et al*., 1997; Goday & Ruiz, 2002; Greciano & Goday, 2006). The paternal genome is eliminated at once *via* the formation of a monopolar spindle (Fig. 5C). This elimination is accompanied by abnormal Н3S10 dephosphorylation, causing the inability of paternal chromosomes to segregate during anaphase and telophase (Escribá *et al*., 2011). Thus, only maternal chromosomes are able to attach to the spindle and segregate properly (Esteban *et al*., 1997; Goday & Esteban, 2001).

### (5) Aberrant decondensation of sperm chromatin caused by PSR and *Wolbachia*

Failure of paternal chromatin decondensation after fertilisation has been observed in organisms that either have a special В chromosome called PSR or are infected with the bacterium *Wolbachia* (Reed & Werren, 1995; Herrick & Seger, 1999; van Vugt *et al*., 2003; Werren *et al*., 2008) (Fig. 6; Table S1). During the first zygotic division, sperm chromatin from males with the PSR chromosome remains compact (van Vugt *et al*., 2003). Phosphorylation of histone H3 and condensin proteins, which ensure formation of metaphase chromosomes, were not detected in the sperm chromatin (Swim *et al*., 2012). Their absence results in the inability of paternal chromatin to form metaphase chromosomes, thus causing elimination of paternal chromatin during the first zygotic division (Swim *et al*., 2012) (Fig. 6A). Only maternal chromosomes, therefore, remain in the egg, leading to haploid organisms that eventually develop into males (van Vugt *et al*., 2003, 2011; Swim *et al*., 2012). PSR avoids this elimination by escaping from the compact paternal chromatin to the maternal chromatin (van Vugt *et al*., 2003, 2011; Swim *et al*., 2012). Abnormal behaviour of the paternal chromatin has been suggested to be triggered by epigenetic modifications established by the PSR chromosome during male gametogenesis (Werren & Stouthamer, 2003; Swim *et al*., 2012). One such possible modification is cytosine methylation, which is detected in the paternal genome during spermatogenesis only in males with the PSR chromosome (Aldrich *et al*., 2017). Moreover, active transcription of the PSR chromosome was detected during male gametogenesis (Akbari *et al*., 2013; Aldrich & Ferree, 2017). A PSR‐linked gene called *haploidizer* expressed during testis development may have a potential role of tagging the paternal chromatin (Dalla Benetta *et al*., 2020).

During *Wolbachia* infection, which causes cytoplasmic incompatibility, sperm chromatin remains compact and unable to decondense (Tram *et al*., 2003, 2006) (Fig. 6B; Table S1). Protamine removal from sperm occurs normally; however, the uploading of histone Н3 is prevented (Landmann *et al*., 2009). As a result, only maternal chromosomes form normally and segregate during anaphase; the paternal genome remains diffuse and lags during anaphase (Landmann *et al*., 2009; Riparbelli *et al*., 2012). A recent study has shown that *Wolbachia*‐expressed deubiquitinases can affect the function of several proteins involved in nuclear import and protamine histone exchange (Beckmann *et al*., 2019; Chen *et al*., 2019). Interestingly, if *Wolbachia* is present, the PSR chromosome is eliminated along with the paternal genome (Reed & Werren, 1995; Werren & Stouthamer, 2003).

### (6) Chromosome lagging and micronuclei formation during genome elimination in plant hybrids

Selective elimination of one of the parental genomes is frequently observed in somatic cell hybrids and interspecific plant hybrids. Such elimination occurs during early development and includes sequential chromosomal loss *via* lagging in anaphase (Bennett *et al*., 1976; Gernand *et al*., 2005, 2006; Houben *et al*., 2011; Sanei *et al*., 2011) (Fig. 7). Elimination of one of the parental genomes results in haploid plant formation. Nevertheless, the ‘rescue’ of such plants can be achieved using artificial manipulations and treatment with microtubule polymerisation inhibitors to restore the diploid chromosomal set, leading to normal diploid non‐hybrid organisms (Kasha & Reinbergs, 1976; Forster & Thomas, 2005; Gernand *et al*., 2006; Ravi & Chan, 2010; Houben *et al*., 2011).

In interspecific plant hybrids, chromosomal lagging can be caused by differences in the timing of crucial mitotic processes, chromosome nondisjunction, which prevents the correct separation of chromatids, or parent‐specific centromere malfunction (Mochida *et al*., 2004; Gernand *et al*., 2006; Ishii *et al*., 2010, 2015*a*,*b*; Sanei *et al*., 2011). In unstable wheat (*Triticum aestivum*) × pearl millet (*Pennisetum glaucum*) hybrid embryos, cohesin bound to the pearl millet chromosomes possibly cannot dissociate normally during cell division, leading to chromosome nondisjunction (Mochida *et al*., 2004; Ishii *et al*., 2010). The specific role of centromeres in selective parental genome elimination has been observed in certain plant species (Ravi & Chan, 2010; Houben *et al*., 2011; Sanei *et al*., 2011). In hybrids between two barley species *Hordeum vulgare* and *H. bulbosum*, centromeric histone CENH3 preferentially uploads to the chromosomes of *H. vulgare*, but not *H. bulbosum*, causing failure of centromere function in the latter (Sanei *et al*., 2011) (Table S1). During early embryonic divisions, genome elimination was not observed, possibly due to residual gamete‐derived *H. bulbosum* CENH3, which provides kinetochore function for the *H. bulbosum* chromosomes. When the amount of gamete‐derived CENH3 falls below a critical threshold, the *H. bulbosum* chromosomes fail to segregate and are eliminated (Sanei *et al*., 2011). Interestingly, in *H. vulgare* × *H. bulbosum*, chromosome elimination is temperature dependent (Pickering, 1985; Sanei *et al*., 2011). Sanei *et al*. (2011) suggested that chaperone proteins can mediate the elimination of *H. bulbosum* chromosomes; however, the process still requires detailed examination (Table S1).

In addition to chromosomal lagging, the budding of micronuclei from the interphase nucleus can eliminate one of the parental genomes in interspecific plant hybrids of wheat × pearl millet (Gernand *et al*., 2005) (Fig. 7; Table S1). Notably, chromatin destined for elimination is spatially separated in the interphase nucleus and localises at the nuclear periphery (Gernand *et al*., 2005). However, the mechanisms leading to genome recognition, followed by chromatin budding, are still unknown.

Both chromosomal lagging and budding cause micronucleus formation, which subsequently leads to heterochromatin modifications and degradation (Gernand *et al*., 2005; Sanei *et al*., 2011). However, rarely, chromosomes enclosed in micronuclei may escape degradation (Tan *et al*., 2015). Such chromosomes can be rescued through nonhomologous end joining, resulting in restructured chromosomes that can be inherited and contribute to increased genetic variability (Tan *et al*., 2015).

### (7) Different pathways of genome elimination in interspecific animal hybrids

Elimination of genetic material often occurs in natural interspecific hybrids which reproduce *via* a hemiclonal pathway (Dawley & Bogart, 1989; Schön *et al*., 2009; Schwander & Oldroyd, 2016; Stöck *et al*., 2021). However, the specific mechanisms of selective genome elimination in these cases are unknown. Even cytological descriptions of the elimination of genetic material have only been performed for a few hybrid forms (Cimino, 1972*a*; Elinson *et al*., 1992; Komaru *et al*., 1998; Zhang, Arai & Yamashita, 1998; Morishima *et al*., 2008; Neaves & Baumann, 2011; Stenberg & Saura, 2013; Zhang *et al*., 2015; Dedukh *et al*., 2020) (Table S1).

During paternal genome elimination in gynogenesis and kleptogenesis, sperm chromatin remains compact, being unable to participate in the first zygotic division (Saat, 1991; Elinson *et al*., 1992; Zhang *et al*., 2015) (Fig. 8A, B). Similarly, in *Drosophila* mutants, in which proteins involved in protamine–histone exchange were affected, sperm chromatin remained compact and underwent subsequent elimination (Loppin, Berger & Couble, 2001; Loppin, Dubruille & Horard, 2015). Homologous proteins involved in protamine–histone exchange were also shown to be inactive in gynogenetic carp (genus *Carassius*) but active in their biological parents (Zhao *et al*., 2011). During gonochoristic reduction in gynogenetic carp, the paternal genome was found to decondense, although it was unable to form metaphase chromosomes, thereby indicating that condensation of paternal chromatin is affected. Failure in the formation of metaphase chromosomes leads to the paternal genome lagging during anaphase, and hence, subsequent elimination (Zhang *et al*., 2015) (Fig. 8B; Table S1).

In interspecific hybrid fishes from the genus *Poeciliopsis* and triploid loaches *M. anguillicaudatus*, elimination of one of the parental genomes usually occurs during mitosis or meiosis (Cimino, 1972*b*; Zhang *et al*., 1998) (Fig. 8E, F; Table S1). Elimination is thought to be related to the inability of chromosomes from one of the parental species to attach to the mitotic or meiotic spindle (Cimino, 1972*b*; Zhang *et al*., 1998). In hybrid fishes from the genus *Poeciliopsis*, monopolar mitotic spindles are formed in germline cells, and only maternal chromosomes can attach to the spindle while paternal chromosomes remain in the cytoplasm (Cimino, 1972*b*). Lagging chromosomes are destined for subsequent degradation (Cimino, 1972*b*; Zhang *et al*., 1998).

Interestingly, in water frog hybrids from the genus *Pelophylax* and carp gudgeons from the genus *Hypseleotris*, elimination of one of the parental genomes occurs gradually during early gametogenesis along with micronucleus formation (Ogielska, 1994; Chmielewska *et al*., 2018; Dedukh *et al*., 2019, 2020; Majtánová *et al*., 2021) (Fig. 8D; Table S1). In water frog hybrids, misaligned and lagging chromosomes have been detected during a series of gonial cell divisions (Ogielska, 1994; Dedukh *et al*., 2019, 2020). Therefore, elimination of one of the parental genomes presumably occurs due to the inability of individual chromosomes to attach to the spindle (Ogielska, 1994; Dedukh *et al*., 2019, 2020). Eliminated chromosomes are enclosed in micronuclei, which accumulate heterochromatin marks and are subsequently degraded *via* autophagy (Chmielewska *et al*., 2018). However, micronucleus formation *via* budding from the interphase nucleus has also been observed in germline cells of water frog hybrids (Chmielewska *et al*., 2018).

## V. CONCLUSIONS

(1) Eliminated genetic material may include chromosomal fragments, whole chromosomes, and even whole parental genomes. Selective elimination of genetic material includes multiple processes that likely evolved independently in different organisms and serve various purposes.

(2) Despite potential differences in mechanisms, selective elimination of genetic material goes through common stages, such as recognition of sequences destined for elimination, epigenetic labelling, spatial separation, physical removal, and final degradation. Elimination often involves the formation of micronuclei, which subsequently are degraded *via* autophagy. Similar processes involved in programmed DNA elimination include its initial sequence‐specific recognition possibly *via* RNA‐dependent mechanisms (scnRNA‐ and piRNA‐dependent elimination in Oligohymenophorea and Spirotrichea ciliates) and tagging of recognised DNA regions *via* heterochromatinisation (DNA methylation and histone modifications).

(3) The removal of sequences is mediated by various mechanisms and usually occurs during mitotic or meiotic divisions; in rare cases, removal of genetic sequences can also occur during interphase. Selective cutting of DNA at specific sites was observed during chromatin diminution. Elimination of whole chromosomes and even whole genomes presumably occurs due to failure of the eliminated chromosomes to attach to the spindle or errors in chromatid segregation. These abnormalities result in chromosomal lagging during anaphase. Chromosome elimination can also occur *via* budding from the interphase nucleus. It results in the enclosure of a portion of chromatin or whole chromosomes in micronuclei, accumulation of double‐strand breaks, and subsequent degradation.

(4) Although many questions in the study of the programmed elimination of genetic material remain unanswered, it is clear that programmed DNA elimination is important for genome plasticity and this may open up new possibilities in research into selection, agriculture, and chromosome or genome editing.

## Supporting information


**Table S1.** Summary of programmed DNA elimination in eukaryotes, the processes involved, their role, distribution among species, ontogenetic stages, types of sequences eliminated and mechanisms of elimination.
