# Supplementary material for: Delete and survive: strategies of programmed genetic material elimination in eukaryotes
Source: Biol Rev Camb Philos Soc. 2021 Sep 20;97(1):195–216. doi: 10.1111/brv.12796 (PMC9292451; doi:10.1111/brv.12796)
Supplement: Supplementary file 1 — Table S1. Summary of programmed DNA elimination in eukaryotes, the processes involved, their role, distribution among species, ontogenetic stages, types of sequences eliminated and mechanisms of elimination. [file BRV-97-195-s001.pdf]

Table S1. Summary of programmed DNA elimination in eukaryotes, the processes, their role, distribution among species, ontogenetic stages, types of sequences eliminated, and mechanisms of elimination. GRC, germline restricted chromosome; HP1, heterochromatin protein 1; INCENP, inner centromeric protein; piRNA, Piwi-interacting RNAs; PSR, paternal sex ratio; scnRNA, scanning RNA.

**Process**	**Distribution**	**Model organisms**	**Cell types**	**Ontogenetic stage**	**Types of sequences eliminated**	**Potential role**	**Mechanism of genetic material elimination**		
							**Recognition**	**Separation (epigenetic modifications)**	**Removal**
Programmed DNA elimination	At least 11 species of parasitic nematodes, 8 species of copepods, lampreys and hagfishes	*Cyclops kolensis*, *C. divulsus*, *C. furcifer*, *C. strenuous*, *Mesocyclops edax*), *Ascaris suum*, *Parascaris univalens*, *Paramyxine* spp., *Eptatretus* spp., *Petromyzon marinus*	Predecessors of somatic cells	Early embryonic development	Tandem repeats, dispersed repeats (retrotransposons), unique (protein coding) sequences	Germ cell *vs* soma cell segregation; genome protection from repetitive elements; gene silencing	unknown	Nematodes: condensation abnormalities, deprivation of CENP-A histone uploading on eliminated regions of their holocentric chromosomes	Cutting before eliminating mitosis; lagging of fragments during anaphase; attachment of telomeres to retaining chromosomes<br><br>Lamprey: eliminated fragments enclosed in micronuclei accumulating heterochromatin modifications (5meC, H3K9me3) followed by degradation
	Two separate classes of ciliates, Oligohymenophorea and Spirotrichea	*Paramecium* and *Tetrahymena*	Developing macronucleus	Formation of new macronucleus			scnRNA recognize sequences destined for elimination	Accumulation of heterochromatin marks (H3K9me3, H3K27me3)	PiggyBac transposase related protein
		Euplotes, *Oxytricha* and *Stylonychia*					piRNA recognise sequences destined for preservation	Accumulation of heterochromatin mark (5meC)	Numerous transposases

Elimination of chromosomes	Some organisms with B chromosomes	B chromosomes in plants (*Aegilops speltoides*)	Somatic cells, specific tissues	Early development	Whole chromosome	Failure of propagation or cell defence mechanisms	unknown		Chromosome nondisjunction, lagging during anaphase
		E chromosomes in sciarid flies (*Sciara ocellaris*, *S. coprophila*)	Somatic cells, germ cells	Early development, meiosis	Whole chromosome	Germ cell and somatic cell segregation; role in gametogenesis	unknown	Semicondensed state, localisation at the nucleus periphery; abnormal dephosphorylation of H3S10P	Failure of chromatid separation, lagging during anaphase; budding from the interphase nucleus of germ cells
		GRC chromosome in passeriform birds (*Taeniopygia guttata*)	Somatic cells, spermatocytes	Early development, meiosis	Whole chromosome	Role in gametogenesis	unknown	H3K9me2, H3K9me3, H4K20me3; during later stages hypophosphorylation of histone H3S10, failure of loading of INCENP, absence of ubiquitination at H2AK119, malfunction of the centromere on GRC; accumulation of double strand breaks	GRCs cannot attach to microtubules of the spindle during metaphase of the first meiotic division and lag in anaphase, forming micronuclei
	Some organisms with XX–X0 sex determination systems such as insects (Sciaridae) and Collembola	Sciarid flies (*Sciara ocellaris*, *S. coprophila*)	Somatic cells, germ cells	Early development	Whole X chromosome(s)	Dosage compensation during sex differentiation	unknown	Abnormal dephosphorylation of H3S10P; semicondensed state, localisation at the nucleus periphery	Failure of chromatid separation, lagging during anaphase; budding from the interphase nucleus of germ cells
	Marsupials from Peramelidae and petaurid families; two eutherian species	*Isoodon*	Somatic cells, specific tissues	Unknown	X or Y chromosomes	Dosage compensation during sex differentiation	unknown	DNA methylation	unknown
Paternal genome elimination	Some arthropod orders with haplodiploid sex determination	Sciarid flies (*Sciara ocellaris*, *S. coprophila*)	Spermatocytes	Spermatogenesis	Whole paternal genome	Dosage compensation during sex differentiation; maternal/patern	unknown	Maternal chromosomes accumulate H3K9ac, H3K14ac, H4K8ac, and	Formation of unipolar spindle leads to elimination of entire paternal genome at once

						al genome competition		H4K12ac; paternal chromosomes accumulate H3K4me2 and H3K4me3; abnormal dephosphorylation of H3S10 in the paternal genome also causes inability to attach to the spindle during anaphase and telophase	
		Lecanoid scale insects (*Planococcus citri*)	Germ cells	Spermatogenesis	Whole paternal genome		unknown	Paternal chromosomes accumulate H3K9me3 and H4K20me3 epigenetic modifications and HP1-related proteins	During inverted meiosis in males maternal and paternal chromosomes assort non-randomly through a monopolar spindle
		Comstockiella scale insects	Germ cells	Spermatogenesis	Whole paternal genome		unknown	Heterochromatinisation early in development	
		Diaspidid scale insects	All cells	Early development in males	Whole paternal genome		unknown	Heterochromatinisation early in development	Paternal chromatids fail to disjoin in an early cleavage division, lag during anaphase and are eliminated
Induced paternal genome elimination	Invertebrates infected by PSR chromosome	PSR infection of *Trichogramma kaykai* and *Nasonia vitripennis*	Zygote	After fertilization	Whole paternal genome	Propagation of parasitic element	unknown	Phosphorylation of histone H3 and condensin proteins that facilitates chromosome condensation not detected in sperm chromatin	Failure of paternal chromatin decondensation; paternal chromatin remains compact and unable to attach to the spindle of the first division of the zygote
	Invertebrates infected by		Zygote	After fertilization	Whole paternal genome	Decreasing number of	unknown	Prevention of uploading H3	Failure of paternal chromatin

	Wolbachia bacteria					uninfected females		histone after removal of protamines from sperm caused by *Wolbachia*-mediated proteins affecting nuclear transport in sperm pronucleus	decondensation, paternal chromatin remains compact and unable to attach to the spindle of the first division of the zygote and lags during anaphase
Parental genome elimination in interspecific plant hybrids	Some interspecific hybrids	*Hordeum vulgare* × *H. bulbosum*	Somatic cells	Early embryonic development	*H. bulbosum* genome		unknown	Upload of centromeric histone CENH3 to the *H. vulgare* chromosomes but not the *H. bulbosum* chromosomes causing the failure of centromere function in *H. bulbosum* genome	Lagging of *H. bulbosum* chromosomes during mitotic division due to disruption of centromere activity
		Triticum aestivum × *Pennisetum glaucum*	Somatic cells	Early embryonic development	*P. glaucum* genome		unknown	Accumulation of heterochromatin modifications after elimination	Chromosomal lagging and budding lead to formation of micronuclei, which are subsequently degraded
Parental genome elimination in interspecific animal hybrids	Kleptogenesis	*Ambystoma*	Zygote	After fertilization	Paternal genome		unknown	Morphologically condensed sperm chromatin	Unknown; paternal chromatin remains compact and unable to attach to the spindle of the first division of the zygote
	Gynogenesis	Hybrids from *Carassius*, *Cobitis* and *Poecilia*	Zygote	After fertilization	Paternal genome		unknown	Morphologically condensed sperm chromatin<br><br>*Carassius* protamine/histone remodelling complex was not observed; absence of H3S10P	Paternal chromatin remains compact and unable to attach to the spindle of the first division of the zygote

								marks	
	Androgenesis	*Corbicula*	Zygote	After fertilization	Maternal genome		unknown	unknown	Maternal chromosomal set extruded *via* polar body formation
	Hybridogenesis	*Pelophylax esculentus*	Germ cells	Gametogenesis	One of the parental genomes		unknown	Accumulation of heterochromatin marks (H3K9me3, H3K27me3) after elimination	Inability of individual chromosomes to attach to the spindle causing their gradual elimination and formation of micronuclei
		Poeciliopsis monach-lucida	Germ cells	Gametogenesis	Paternal genome		unknown	unknown	Formation of unipolar mitotic spindle; only maternal chromosomes can attach to the spindle while paternal chromosomes remain in the cytoplasm
		Triploid hybrid fish *Misgurnus anguilicaudatus*	Oocyte	First meiotic division	One of the parental genomes		unknown	unknown	Formation of bivalents and univalents; only bivalents are able to attach to the spindle; univalents remain in the cytoplasm during anaphase of meiosis
